# Influence of Room Illumination on Corneal Densitometry

**DOI:** 10.1167/tvst.15.10.2

**Published:** 2026-10-05

**Authors:** Miriam Esteban-Burgués, Ana R. Arizcuren, Cristina Pelayo, Alejandra Consejo

**Affiliations:** 1 Aragon Institute for Engineering Research, Universidad de Zaragoza, Zaragoza, Spain

**Keywords:** corneal densitometry, Scheimpflug imaging, optical coherence tomography, illumination, corneal transparency

## Abstract

**Purpose:**

To evaluate the effect of room illumination on corneal densitometry measurements obtained with Scheimpflug imaging and optical coherence tomography (OCT) and on corneal geometric parameters.

**Methods:**

Twenty-five healthy participants (50 eyes) were imaged under four controlled illumination conditions: extreme scotopic (0.003 lx), dim-light/mesopic (30 lx), low photopic (130 lx), and moderate photopic (220 lx) conditions. Corneal densitometry was quantified as the mean pixel intensity for both the Scheimpflug and the OCT images, with speckle contrast additionally computed for the OCT images. Corneal geometry (central corneal thickness and keratometry readings defined as corneal power in the flattest [K1] and steepest [K2] meridians) was extracted from Scheimpflug tomography. Linear mixed-effects models were used to assess the effect of illumination on corneal parameters.

**Results:**

Scheimpflug-derived corneal densitometry increased significantly with illumination (*P* < 0.001), rising from 45.8 ± 4.3 arbitrary units under extreme scotopic conditions to 69.5 ± 11.5 arbitrary units under moderate photopic illumination. Significant differences were observed between all illumination levels (all *P* < 0.01). In contrast, the OCT-derived mean pixel intensity and speckle contrast remained stable across conditions (*P* > 0.05). The corneal geometric parameters showed no significant changes with room illumination (all *P* > 0.05).

**Conclusions:**

Scheimpflug-based corneal densitometry is highly sensitive to room illumination, even within clinically plausible lighting conditions, whereas the OCT-derived metrics are robust to such variations.

**Translational Relevance:**

Room illumination-induced changes may bias corneal densitometry readings and should be carefully controlled and reported in densitometry studies.

## Introduction

Corneal densitometry is gaining increasing interest as a tool for objective corneal tissue characterization in both clinical and research settings.^1^ By providing quantitative information on corneal transparency, it has shown potential for detecting the subtle changes associated with conditions such as keratoconus,^2^^–^^5^ refractive surgery,^6^^,^^7^ or contact lens wear.^8^^,^^9^ Corneal densitometry is typically assessed using Scheimpflug imaging; more recently, optical coherence tomography (OCT) has been explored as an alternative modality for corneal tissue characterization.^10^

However, densitometry metrics derived from image-based systems are known to be influenced by several confounding factors unrelated to intrinsic tissue properties. These include age,^11^ anterior segment biometry,^12^ iris pigmentation,^13^ spurious brightness from adjacent ocular structures,^14^ and variations in eye alignment during image acquisition.^15^ As a result, measured changes in densitometry may not exclusively reflect true alterations in corneal transparency.

Among these factors, the potential influence of examination room illumination (hereafter referred to as “room illumination”) has received little attention. In clinical practice, ophthalmic imaging is often performed under varying lighting conditions, and there is currently no specific standardization for illumination in ophthalmic imaging rooms. Although general indoor lighting is often regulated by regional standards (e.g., EN 12464-1:2021 in Europe and its national implementations, such as UNE-EN 12464-1:2022 in Spain), these standards are not tailored to ophthalmic imaging conditions, potentially introducing an additional, yet unquantified, source of bias in densitometry measurements.

The aim of this study was to evaluate the effect of controlled room illumination on corneal densitometry measurements obtained with Scheimpflug imaging and OCT imaging and to assess whether these effects also influence corneal geometric parameters.

## Methods

### Data Collection

This study was approved by the Ethics Committee for Clinical Research of Aragon (PI25/184) and adhered to the tenets of the Declaration of Helsinki. All participants provided written informed consent to participate after the nature and possible consequences of the study had been explained.

Twenty-five healthy Caucasian participants (10 men and 15 women) were included (mean age, 25.0 ± 4.8 years), providing measurements from 50 eyes. The sample size was estimated based on the expected primary outcome, namely, Scheimpflug-derived corneal densitometry. Assuming a paired repeated-measures design, a two-sided significance level of 0.05, and 90% statistical power, sample size estimation was based on a previous Scheimpflug corneal densitometry study reporting that differences of only a few arbitrary units (a.u.) may already distinguish healthy from subclinical pathological corneas, with reported standard deviations between 2 and 5 a.u.^3^ Accordingly, the present study was designed to detect differences of approximately 4 a.u. between illumination conditions. Under these assumptions, the estimated minimum sample size was 17 participants. To provide a margin for potential unusable acquisitions or missing data, although no such cases ultimately occurred, 25 participants were ultimately recruited. All participants showed normal visual function, as confirmed by best-corrected visual acuity assessment. All participants underwent slit-lamp examination before imaging. Only eyes with a clinically normal tear film and no signs of ocular surface disease were included in the study. No fluorescein or other ocular surface dyes were instilled before image acquisition. Eyes included in the study had no history of ocular surgery or corneal pathology, showed no signs of senile arcus, and had not worn contact lenses for at least 48 hours before the examination; additionally, none of the participants were rigid gas-permeable lens wearers.

All measurements were conducted in a controlled laboratory setting at the University of Zaragoza. Scheimpflug imaging was performed using a Pentacam HR tomographer (OCULUS Optikgeräte GmbH, Wetzlar, Germany). Corneal densitometry was quantified as the corneal mean pixel intensity (MPI), calculated from the exported Scheimpflug images. In addition, corneal tomographic parameters including central corneal thickness (CCT) and keratometry readings defined as corneal power in the flattest meridian (K1) and corneal power in the steepest meridian (K2) were obtained directly from the Pentacam HR software output. In addition, OCT imaging was performed using a spectral-domain OCT system REVO FC 130 (Optopol Technology, Zawiercie, Poland) and was also used to assess corneal densitometry.

### Lighting Conditions and Acquisition Protocol

All measurements were performed under controlled room illumination conditions. Ambient illumination was set to four predefined levels covering clinically plausible lighting environments: extreme scotopic darkness (I1, 0.003 lx), dim-light/mesopic condition (I2, 30 lx), low photopic illumination (I3, 130 lx), and moderate photopic illumination, representative of a clinical examination room (I4, 220 lx), where I1–I4 denote illumination levels 1 to 4, respectively. These illumination levels were selected to cover the range typically encountered in ophthalmic examination rooms. To achieve the extreme scotopic condition (I1), reduction of ambient room lighting alone (lights off and window blinds closed) was insufficient. Therefore, participants were additionally covered with an opaque blackout drape provided by the device manufacturer.

Room illumination was provided by three LED light strips equipped with diffusers (color temperature 6000 K) mounted on the ceiling above the imaging devices and directed downward toward the examination area. This configuration was selected to reproduce the overhead illumination geometry typically encountered in clinical examination rooms while providing homogeneous ambient illumination across the measurement area. The illumination intensity was continuously monitored using a calibrated silicon photodiode (BPW21R, Vishay Semiconductors, Malvern, PA) with a sensitivity of 9 nA/lx, placed at a fixed position in the same horizontal plane as the participant's eye and adjacent to the entrance pupil of the imaging devices (within approximately 50 cm). The BPW21R photodiode was selected because, according to the manufacturer's specifications, it is designed for high-precision linear applications and exhibits high linearity over a wide range of illumination levels. As a part of the experimental setup, the illumination was verified to be spatially uniform across the measurement plane by sampling multiple locations at eye level. The photodiode current was converted into a voltage signal using a transimpedance amplifier (AMP100, Thorlabs, Newton, NJ) with a gain of 10 MV/A. The illumination levels were, therefore, recorded as voltage values, which were subsequently converted into illuminance (lx) using the known photodiode sensitivity and amplifier gain, enabling the accurate quantification of the room illumination, particularly under low light conditions.

For each participant, measurements were acquired sequentially under all four illumination conditions following a predefined protocol. Illumination levels were applied in a fixed order, from I4 to I1, and imaging was consistently performed first with the Pentacam HR Scheimpflug tomographer and subsequently with the OCT REVO FC 130 to ensure a standardized and reproducible acquisition procedure across participants. At each illumination level, three repeated measurements were obtained per eye and per imaging modality (Scheimpflug and OCT).

### Image Processing and Corneal Parameters

All images were processed using custom-written routines in MATLAB (MathWorks, Inc., Natick, MA, version R2024b). Both the Scheimpflug and OCT systems provide grayscale images, with pixel intensity values ranging from 0 (black) to 255 (white). The Scheimpflug images had a fixed size of 1200 × 620 pixels, and the OCT corneal B-scans had a fixed size of 1280 × 992 pixels. In both modalities, the acquired images represent the anterior segment of the eye, including the full cornea, the iris, and the anterior portion of the crystalline lens.

For both imaging modalities, corneal densitometry was quantified as the corneal MPI, defined as the arithmetic mean of pixel intensity values within a segmented corneal region of interest (ROI). In all cases, the ROI was defined in physical space and laterally restricted to the central 10 mm of the cornea for both the Scheimpflug and OCT images, while its axial thickness corresponded with the full corneal thickness of each individual eye, as determined from the segmented anterior and posterior corneal boundaries. This lateral restriction was applied to minimize the influence of the peripheral spurious brightness commonly observed in Scheimpflug images near the corneoscleral junction and the adjacent scleral regions, as previously reported,^14^ while ensuring consistency of the analyzed region across the imaging modalities.

When illumination-dependent changes in the corneal densitometry metrics were identified, background intensity was additionally quantified to help interpret whether these changes could be related to overall image brightness conditions. The background MPI was computed from a predefined noncorneal ROI located in the upper portion of the image, free from anatomical structures. Representative images and examples of the ROIs used for corneal densitometry and background analysis are shown in Figure 1.

**Figure 1.**
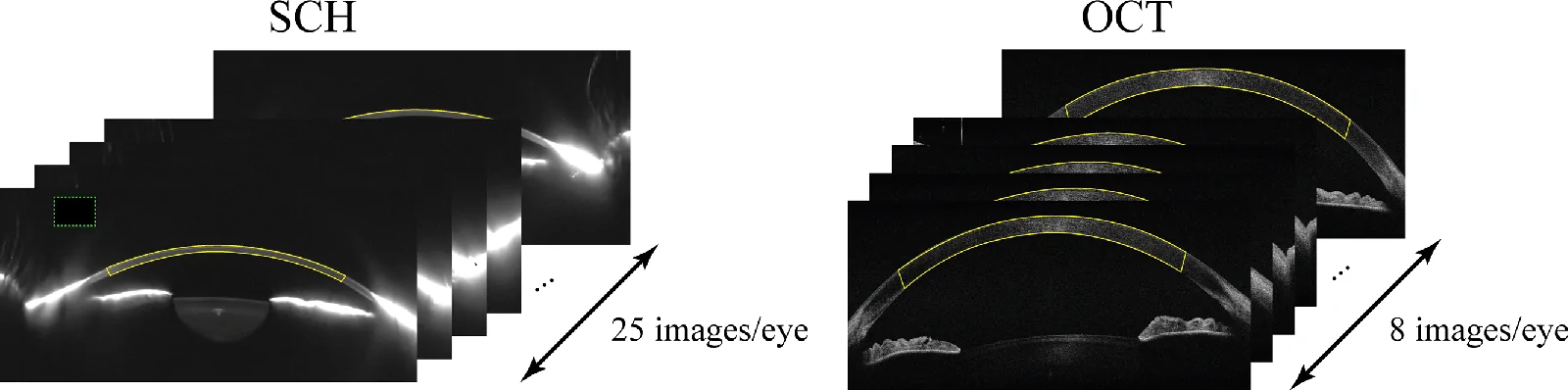
Representative Scheimpflug (SCH) and OCT images illustrating the image acquisition strategy. For each eye, 25 SCH meridional images and 8 OCT radial B-scans were acquired. Automatic segmentation was applied to identify the corneal ROI (*yellow contours*), from which the corneal densitometry was calculated. The *green dashed box* in the SCH image illustrates the region used for background quantification.

#### Scheimpflug Imaging

Each Scheimpflug acquisition provided 25 meridional images evenly distributed over 360°. Automatic segmentation was applied independently to each meridian to isolate the corneal tissue from the surrounding structures, including the iris, crystalline lens, and peripheral glare regions, as described in detail in previous work.^14^ Briefly, a median filter was applied to reduce noise, followed by edge enhancement and Canny-based edge detection to identify the anterior and posterior corneal boundaries. These boundaries were used to generate a binary mask defining the corneal ROI described elsewhere in this article and illustrated in Figure 1 (left). The MPI was computed for each meridional image, and the values were averaged across the 25 meridians to obtain a single representative corneal densitometry value per eye and illumination condition.

In addition to image export, the corneal geometric parameters were obtained directly from the Pentacam HR software output, including the CCT, K1, and K2. These parameters are automatically calculated from the device's three-dimensional tomographic reconstruction following the instrument's standard alignment procedure and were used to assess the potential influence of the room illumination on the corneal geometry.

#### OCT Imaging

OCT corneal imaging was performed in radial scan mode, consisting of eight meridional B-scans centered on the corneal apex. For each B-scan, automatic segmentation was applied to detect the anterior and posterior corneal interfaces. Before segmentation, contrast-limited adaptive histogram equalization and Gaussian filtering were used to enhance boundary visibility and reduce peripheral noise. The corneal ROI was defined using the same criteria described elsewhere in this article and is illustrated in Figure 1 (right). The MPI values were computed for each B-scan and averaged across the eight meridians to obtain a single corneal MPI estimate per eye and illumination condition.

In addition to the MPI, speckle contrast was computed for the OCT images as the ratio between the standard deviation of the pixel intensity and the mean of the pixel intensities within the corneal ROI, following the standard definition used in coherent imaging. This metric was not computed for the Scheimpflug images, because speckle is not present in incoherent illumination systems.

### Statistical Analyses

Statistical analyses were performed using MATLAB. The effect of room illumination on the corneal densitometry and geometric parameters was evaluated using linear mixed-effects models to account for the repeated-measures structure of the data.

For densitometry outcomes, separate models were fitted for each imaging modality. For Scheimpflug imaging, the corneal MPI was analyzed, whereas for OCT imaging both the MPI and speckle contrast were considered. For corneal geometry, the CCT, K1, and K2 extracted from Scheimpflug tomography were analyzed independently. In all models, the room illumination was included as a fixed effect with four levels (I1–I4). Subject was included as a random intercept, and eye was included as a random intercept nested within subject. This method accounted for the correlation among repeated measurements obtained from the same eye, while also modeling the correlation between fellow eyes from the same participant. When a significant main effect of illumination was detected, post hoc pairwise comparisons between illumination levels were performed with an appropriate correction for multiple comparisons. Statistical significance was set at a *P* value of less than 0.05.

In addition, within-condition repeatability was assessed using the coefficient of variation (CV), calculated from repeated measurements acquired under identical illumination conditions. Data are reported as mean ± standard deviation unless otherwise stated.

## Results

Room illumination produced a clear effect on the corneal densitometry derived from Scheimpflug imaging, whereas no corresponding effect was observed in OCT-based metrics. This modality-dependent behavior is evident on visual inspection of Figure 2, which shows representative corneal images from one randomly selected subject acquired under increasing room illumination levels. The Scheimpflug images exhibit a progressive increase in the apparent corneal brightness with illumination, while the corresponding OCT B-scans show no appreciable changes in the corneal appearance or the speckle pattern.

**Figure 2.**
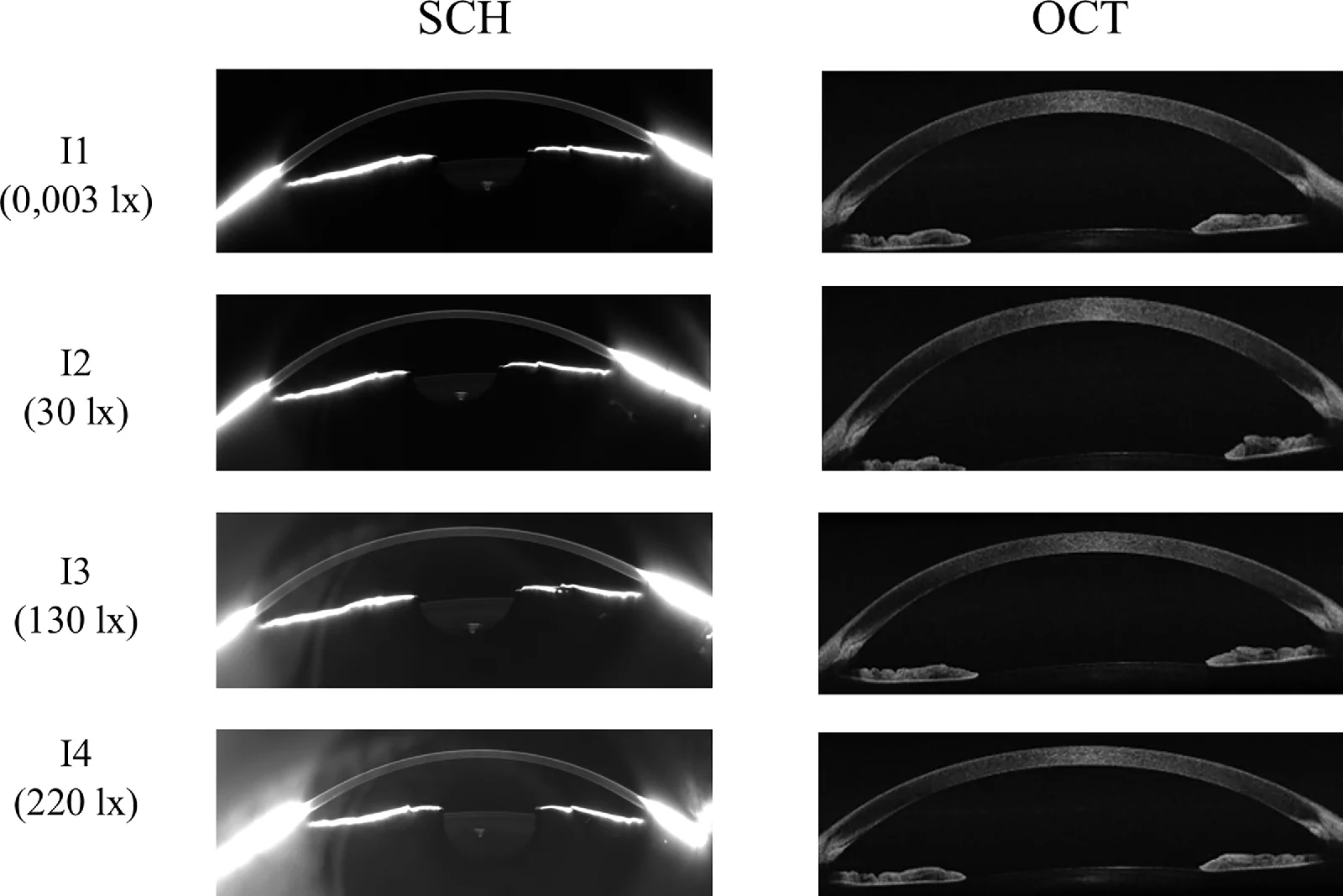
Representative corneal images from one subject acquired under increasing room illumination levels (I1–I4). Scheimpflug images (SCH, *left column*) show progressive increases in apparent corneal brightness with illumination, whereas the corresponding OCT B-scans (*right column*) exhibit no visible changes in the corneal appearance or the speckle structure.

The quantitative results are summarized in Figure 3. The Scheimpflug corneal MPI (Fig. 3, left) showed a monotonic increase from 45.8 ± 4.3 a.u. under extreme scotopic conditions (I1) to 69.5 ± 11.5 a.u. under moderate photopic illumination (I4), with intermediate values of 49.8 ± 5.4 a.u. (I2) and 60.7 ± 8.4 a.u. (I3). Linear mixed-effects modeling revealed a highly significant effect of room illumination on the Scheimpflug-derived corneal MPI (*P* < 0.001). Post hoc pairwise comparisons showed statistically significant differences between all illumination levels (all *P* < 0.01), indicating that even moderate changes in room illumination lead to measurable variations in the Scheimpflug-based corneal densitometry.

**Figure 3.**
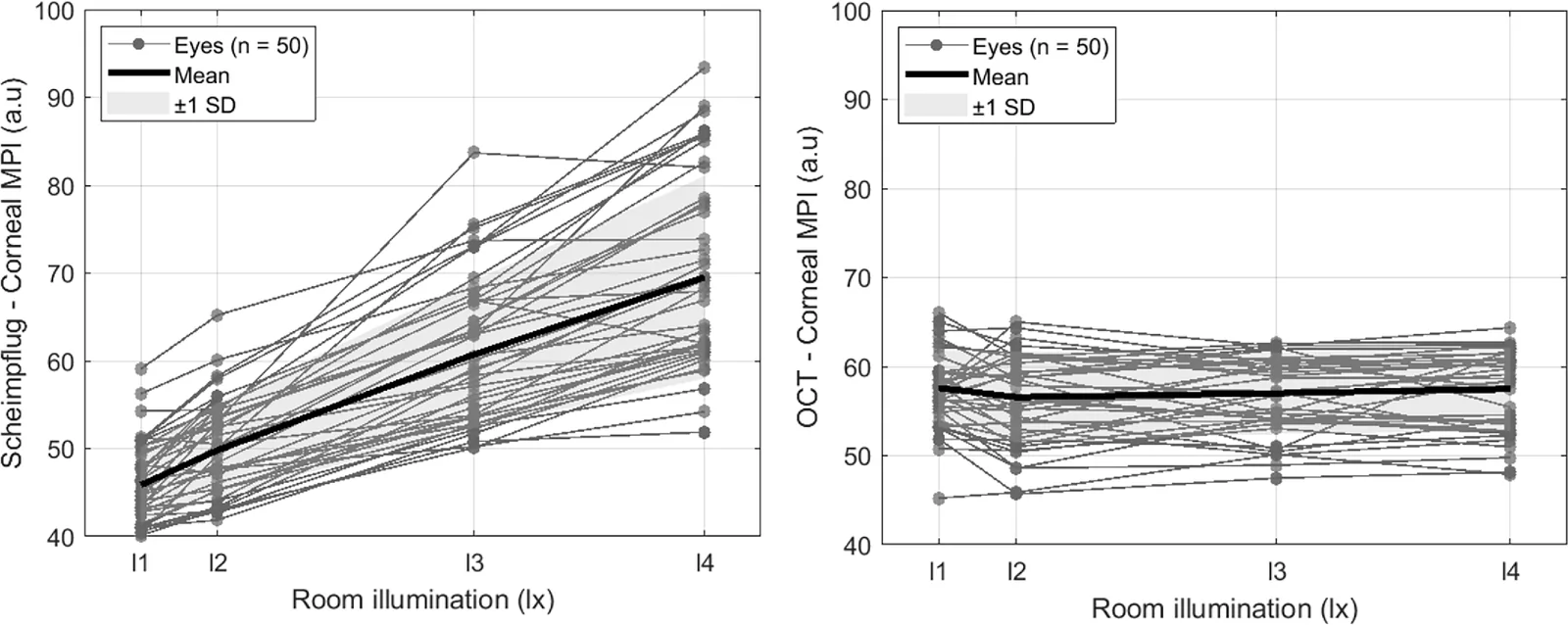
The effect of room illumination on corneal densitometry, calculated as the MPI measured with Scheimpflug imaging (*left*) and OCT imaging (*right*). Individual eye data, averaged across the three repeated measurements obtained under each illumination condition, are shown together with group mean ± standard deviation (SD). I1, 0.003 lx; I2, 30 lx; I3, 130 lx; and I4, 220 lx.

In contrast, the OCT-derived corneal MPI remained stable across the illumination conditions, with overlapping distributions across all levels (Fig. 3 right). The mixed-effects model revealed no significant effect of illumination on the OCT MPI (*P* > 0.05). Similarly, OCT speckle contrast did not exhibit significant changes with illumination (*P* > 0.05).

Additionally, a repeatability analysis confirmed low variability across repeated measurements of corneal densitometry metrics. For Scheimpflug imaging, the CV of the corneal MPI remained below 0.5% across all the illumination conditions, indicating excellent repeatability. For the OCT-derived metrics, CV values were below 5% for both the MPI and speckle contrast, consistent with the good repeatability of OCT-based measurements.

As illumination-dependent changes in the Scheimpflug corneal MPI were identified, the background intensity was analyzed. The background MPI increased with room illumination and showed a strong linear association with the corneal MPI when the mean values for each illumination condition were considered (*R*^2^ = 0.997; larger markers in Fig. 4). Individual measurements (smaller markers in Fig. 4) exhibited greater scatter and did not follow a single narrow linear relationship. Additionally, the dispersion of the corneal MPI values for a given background intensity increased with the illumination level (Fig. 4).

**Figure 4.**
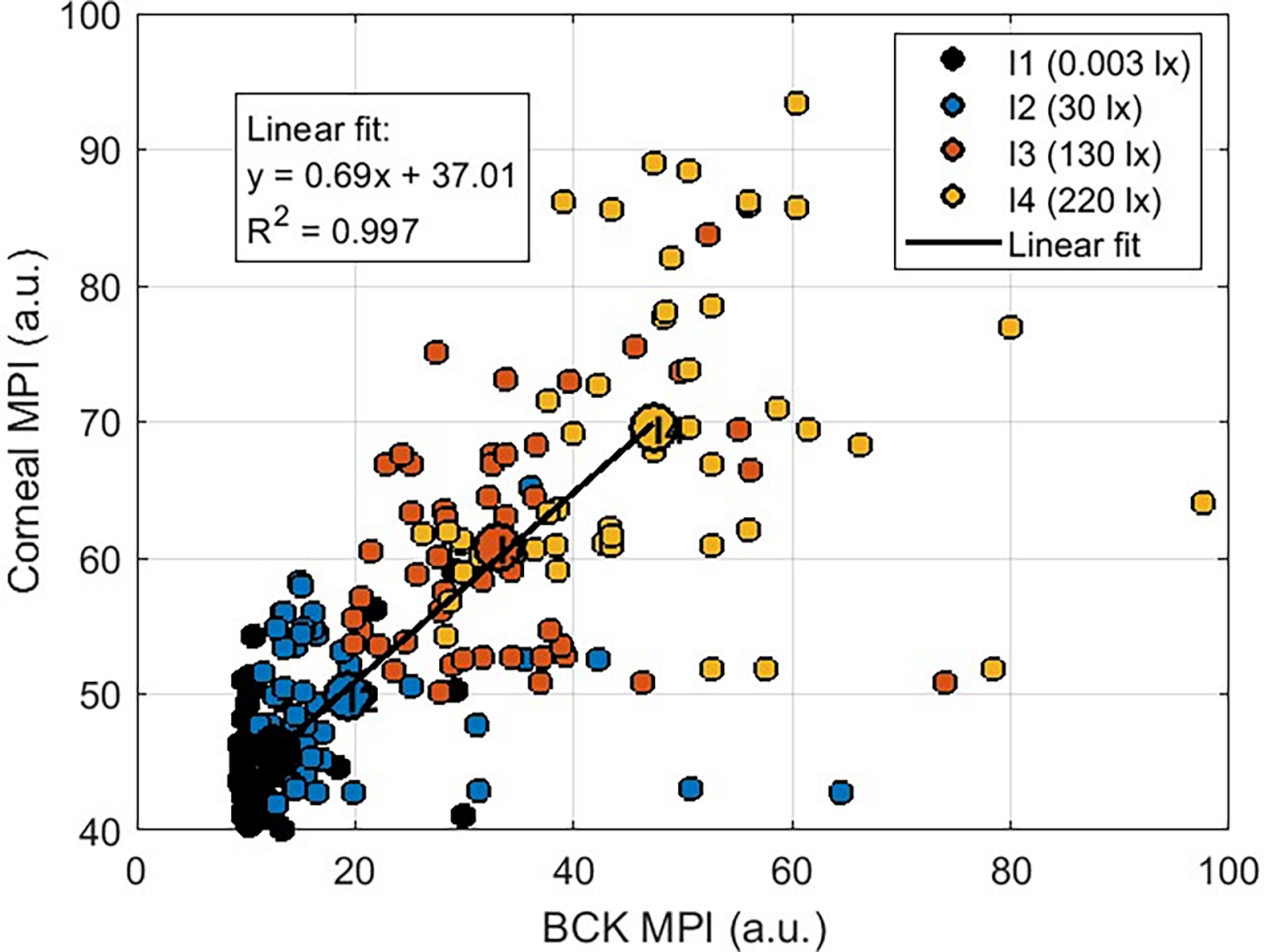
Relationship between the corneal MPI and the background MPI (BCK) in Scheimpflug imaging across the illumination conditions. *Small markers* represent individual eye measurements, color-coded according to illumination level (I1–I4). *Large markers* indicate the corresponding group mean values for each illumination condition. The *black line* represents the linear regression fitted to the four group mean values.

Finally, the Scheimpflug-derived corneal geometric parameters, including the CCT, K1 and K2, showed no significant dependence on room illumination (Table 1, all *P* > 0.05), confirming that illumination selectively affects densitometry-related metrics while corneal geometry remains stable.

**Table 1.** Scheimpflug-Derived Corneal Geometric Parameters (CCT, K1, K2) Across Illumination Conditions (I1–I4)

Illumination Condition	CCT (µm)	K1 (D)	K2 (D)
I1 (0.003 lx)	534 ± 31	41.97 ± 1.47	43.26 ± 1.53
I2 (30 lx)	535 ± 31	41.99 ± 1.45	43.27 ± 1.52
I3 (130 lx)	534 ± 31	41.97 ± 1.46	43.44 ± 2.03
I4 (220 lx)	534 ± 30	41.99 ± 1.48	43.28 ± 1.52
*F* statistics	*F*(3,144) = 0.48	*F*(3,144) = 1.06	*F*(3,144) = 0.67
*P* value	*P* = 0.69	*P* = 0.36	*P* = 0.57

Values reported as mean ± SD. *P* values are from linear mixed-effects models.

## Discussion

This study demonstrates that corneal densitometry derived from Scheimpflug imaging is highly sensitive to room illumination, within illumination ranges commonly encountered in routine clinical practice, whereas OCT-based corneal intensity and speckle descriptors remain largely unaffected (Figs. 2 and 3). Importantly, these illumination-dependent effects were confined to densitometry-related image descriptors and did not affect corneal geometric parameters (Table 1).

The magnitude of the illumination effect observed in Scheimpflug densitometry is clinically relevant. Across the illumination range tested, the corneal MPI increased from approximately 45.8 a.u. under extreme scotopic conditions (I1) to nearly 69.5 a.u. under moderate photopic illumination (I4), representative of a clinical examination room, corresponding with an apparent increase of approximately 50% to 55%. Such a change does not reflect a true alteration in corneal tissue transparency but rather a change in image brightness driven by the acquisition conditions. Consequently, variations in room illumination may create the appearance of an altered corneal transparency, potentially leading to the misinterpretation of densitometry results. Importantly, a repeatability analysis confirmed that the observed illumination-dependent changes were substantially larger than measurement variability (CV < 0.5% for the Scheimpflug images). These findings support that the illumination-induced changes observed in Scheimpflug densitometry are not driven by measurement noise but reflect a systematic effect related to the acquisition conditions.

This finding is particularly important given the increasing interest in corneal densitometry as a tool for the early or subclinical detection of corneal diseases, such as keratoconus,^2^^,^^3^ where the expected differences between normal and subclinical cases are subtle. For example, previous work has reported mean densitometry-related values of approximately 39 ± 3 a.u. in control eyes and 43 ± 4 a.u. in subclinical keratoconus, corresponding with differences on the order of only a few intensity units.^3^ In this context, illumination-induced changes of the magnitude observed here could plausibly contribute to false-positive or false-negative interpretations, especially when comparing measurements acquired under different lighting conditions or at different clinical sites.

An additional relevant finding is that statistically significant differences in the Scheimpflug corneal MPI were detected not only between extreme illumination conditions but also between illumination levels that may appear to be subjectively similar to the examiner. Notably, these differences were observed between the mesopic condition (I2) and the extreme scotopic conditions (I1), despite I2 appearing visually dark in the examination room. Achieving true extreme scotopic conditions required not only turning off the room lights and closing the window blinds, but also shielding participants with an opaque blackout drape. This finding highlights that perceived darkness is not equivalent to controlled low illuminance and that consistency in the illumination level is critical when longitudinally monitoring densitometry-related metrics.

The background intensity analysis further supports an illumination-related origin of the observed Scheimpflug densitometry changes. The background MPI increased systematically with room illumination and was strongly correlated with the corneal MPI when considering the average values obtained for each illumination condition (Fig. 4). In contrast, the individual measurements exhibited an increasing dispersion at higher illumination levels (yellow markers in Fig. 4), indicating that illumination does not act as a simple uniform offset across participants. Instead, the observed variability suggests an interaction between the illumination conditions and subject- or acquisition-specific factors. These factors, as reported in the literature, may include anterior segment biometry,^12^ peripheral spurious brightness from adjacent structures such as the sclera,^14^ iris pigmentation,^13^ or variations in eye alignment relative to the imaging axis.^15^ The background intensity therefore provides useful contextual information about the acquisition conditions, rather than serving as a corrective factor.

The different behavior observed between Scheimpflug imaging and OCT can be explained by fundamental differences in image formation. Scheimpflug systems rely on incoherent illumination and camera-based detection, making the recorded pixel intensities inherently susceptible to ambient light entering the optical path or influencing the camera response.^16^ In contrast, OCT is based on coherent detection with an internal reference arm, and image formation depends on interference rather than absolute intensity levels.^17^ As a result, the OCT-derived intensity and speckle metrics are largely insensitive to changes in room illumination, as confirmed by the stability of both the MPI and the speckle contrast across all the tested conditions. Notably, OCT has previously been used to study corneal tissue-related changes reported with aging,^18^^,^^19^ epithelium integrity,^20^ or intraocular pressure,^21^ supporting its sensitivity to biologically meaningful variations in scattering and microstructure. Although OCT has not yet been widely adopted for routine clinical corneal densitometry, its intrinsic robustness to ambient lighting conditions, combined with its greater spatial resolution and its ability to capture tissue-dependent changes,^22^ suggests that it could be a valuable tool for corneal densitometry studies.

Regarding illumination measurement, a conventional lux meter was not used because such devices typically lack sufficient resolution and sensitivity under very low light conditions. The accurate characterization of extreme scotopic darkness required resolution on the order of at least three decimal places, which standard lux meters (calibrated for measuring light rather than near darkness) do not provide.^23^^,^^24^ The photodiode-based approach used in this study enabled the precise and reproducible quantification of illumination across the full tested range. Importantly, the illumination conditions were deliberately restricted to levels representative of realistic clinical imaging environments, spanning the extreme scotopic darkness to the moderate photopic illumination typically encountered in clinical examination rooms. Natural daylight conditions were not considered, because corneal imaging in clinical practice is usually performed under controlled lighting, often semidark or moderately photopic, rather than under variable ambient daylight.

It should also be noted that the MPI is a device-specific descriptor that reflects apparent image brightness rather than a directly comparable physical measure across the imaging modalities. In the case of OCT, the MPI is strongly influenced by system-dependent signal scaling, sensitivity roll-off, and depth-dependent attenuation and therefore does not directly represent absolute tissue-scattering properties.^25^ For this reason, the MPI alone may not be optimal for quantitative tissue characterization in OCT imaging.^18^ In contrast, speckle contrast, which normalizes the local intensity variability by the mean signal level, is more closely related to the tissue microstructure and is less dependent on absolute signal scaling. In the present study, both the OCT-derived MPI and speckle contrast remained stable across illumination conditions, supporting the interpretation that the OCT-based metrics are largely insensitive to ambient light variations. The present work did not aim to compare absolute densitometry values between imaging modalities; such comparisons would require dedicated normalization and calibration strategies and are beyond the scope of this study, but represent an interesting direction for future work.

Several limitations should be acknowledged. First, pupil size may have contributed to the observed corneal densitometry values. It is well known that pupil diameter varies with illumination level,^26^ which may affect the apparent corneal densitometry.^12^^,^^14^ In particular, changes in pupil size can alter the amount of visible iris and its associated reflectivity, potentially impacting the measured signal.^13^ However, in the present study, pupil-related effects are expected to be limited, because the illumination conditions, although controlled, did not induce extreme pupil responses. This assumption is qualitatively supported by the representative images shown in Figure 2, where no marked changes in pupil size are observed across the differing conditions. Future studies could further isolate this effect by pharmacologically controlling pupil diameter (e.g., by using mydriatic agents). Additionally, the study was conducted in a cohort of young, healthy participants, and the findings may not directly extrapolate to pathological corneas, where scattering properties and signal-to-noise characteristics differ. Furthermore, only one Scheimpflug and one OCT system were evaluated, and illumination sensitivity may vary across devices and manufacturers.

From a methodological perspective, these results emphasize the importance of explicitly reporting and controlling room illumination in studies using Scheimpflug-based corneal densitometry, in accordance with previous work.^27^ Future densitometry studies should incorporate illumination conditions into their acquisition protocols and reporting standards to ensure comparability across visits, subjects, and clinical sites.

## Conclusions

Scheimpflug-based corneal densitometry is strongly influenced by room illumination, even within clinically routine lighting conditions, whereas OCT-derived corneal densitometry and speckle metrics are largely robust to such variations. The careful control and documentation of illumination conditions are therefore essential to avoid the misinterpretation of densitometry findings and to ensure that the observed changes truly reflect corneal tissue properties rather than acquisition-related artifacts.
